# Randomized effectiveness Trial of the Family Check-Up versus Internet-delivered Parent Training (iComet) for Families of Children with Conduct Problems

**DOI:** 10.1038/s41598-018-29550-z

**Published:** 2018-07-31

**Authors:** Ata Ghaderi, Christina Kadesjö, Annika Björnsdotter, Pia Enebrink

**Affiliations:** 1 Karolinska Institutet, Department of Clinical Neuroscience, Division of Psychology, Stockholm, Sweden; 2 University of Gothenburg, Sahlgrenska Academy, Gillberg Neuropsychiatry Centre, Gothenburg, Sweden; 3 University of Gothenburg, Department of Psychology, Gothenburg, Sweden

## Abstract

We investigated the effectiveness of the Family Check-Up (FCU) and an Internet-based parent-training program (iComet), along with moderators and mediators of outcome. Families (N = 231) with a child with conduct problems were randomized to one of the conditions for 10 weeks of treatment. The drop-out rate was significantly higher in the iComet (39%) compared to FCU (23%). At post-treatment, both conditions resulted in significant improvement, based on parent-report, but no significant interaction between time and condition, with the exception of conduct problem subscale of the Strengths and Difficulties Questionnaire, slightly favoring the FCU. Neither child, nor teacher reports indicated any significant changes on any of the investigated variables. At 1-, and 2-years follow-up, the gains from the treatment were maintained in both conditions, with basically no significant time X condition interactions. A significantly larger proportion of children in the FCU recovered at post-treatment with regard to opposition defiant behavior, inattention, and conduct problems, compared to the iComet, but almost none of these differences remained significant at 1-, and 2-years follow-up. None of the moderators (child age, parental income or education, or pre-treatment level of motivation) or mediators (limit setting, and appropriate or harsh parenting) of outcome turned out to be significant.

## Introduction

The efficacy of a significant number of parent-based interventions for prevention or treatment of externalizing problems has been investigated in a large number of studies, and summarized in systematic reviews and meta-analyses^1–3^. Evaluations of pre- to post- measurement changes generally show small to moderate effect sizes^1,4,5^. Most of these evaluations are based on efficacy trials (explanatory trials), which evaluate whether the intervention is effective under ideal conditions, as compared to effectiveness trials (pragmatic trials), where the intervention is evaluated in clinical settings more similar to the “real world”. A systematic review and meta-analysis^6^ suggested that the short-term effects of parent-based interventions such as parent-training programs do not differ significantly in efficacy and effectiveness trials, respectively. However, we have restricted knowledge about the long-term effects of these programs^1^. Follow-up-periods beyond 6 months including both the intervention group and a control condition are scarce^7^. There are a few long-term follow-ups of randomized effectiveness trials of parent training programs available^8^, but the majority are efficacy trials with the interventions delivered by well trained research staff and compared to no-treatment wait-list control groups.

In comprehensive overviews of interventions for children and youth with externalizing behavior problems^9^, some of the parent-training programs listed as evidence-based (well-established or probably efficacious) are the Parent-Management Training-Oregon model^10^, the Incredible Years^11^, Positive Parenting Programs Triple P^12^, and Parent-Child Interaction Therapy^13^ (for a review see^14^). According to a meta-analysis on prevention programs^7^ and the Blueprints for Healthy Development and SAMHSA’s National Registry of Evidence-based Programs and Practices, a family-based program, the Family Check-Up (FCU^15^), is also described to have a good evidence-base. The FCU is a second generation of parent training programs^16–19^, with effects on reductions of externalizing behavior problems^20^, substance use^18^, and prevention of child depression^21^. The FCU is based on the Oregon PMT-model but has been designed to be ecologically more valid as it is tailored to the needs and motivation of families, grounded in a structured assessment and feedback phase during the first three sessions. Long-term evaluations of the FCU have shown reductions of externalizing behavior in children compared to a control group at 3 and 4 years of age (12 and 24 month follow-ups)^15^, and the FCU has also been found to reduce problem behaviors, arrests, alcohol, tobacco use among high-risk adolescents from 11 years of age, up to age 17^22^.

Despite the current state of evidence, a majority of families with children in need of services due to psychiatric disorders do not have access to evidence-based treatments^23^. For families who start an intervention, the dropout rate is between 40–60%^23^. Existence of evidence-based interventions thus does not automatically translate into improved public health. Future interventions need to include features that enhance access to treatments, both for already established problems but also in order to prevent problems from escalating. Future interventions also need to improve retention rates. As argued by leading researchers in the field^24^, we may have very limited success in decreasing the prevalence and incidence of psychiatric and psychological problems without a major shift and expansion in clinical practice and intervention research. Use of modern technology such as the Internet might be a viable option to increase access to psychological treatments, also to enable flexible use of interventions before problems become severe. During the past few years, parent-training programs for externalizing problems have been adapted for delivery through the Internet with secured web-based support^25–28^. For instance, the Triple P^29^ was translated into an online version for parents of children with externalizing problems, showing reduced child behavior problems as well as improved parenting competences when evaluated in a randomized controlled trial. The Swedish parent-training program Comet^30^, similarly highly influenced by principles in the Parent-Management Training- Oregon Model^10^ and the Incredible Years model^11^, was also adapted for delivery through Internet (“iComet”) and made available to parents with children with externalizing problems^25^. Parents in the iComet reported more reduced child behavior problems and improved parenting skills, compared to parents on a waitlist control condition. A commonality for Internet-based interventions is that they are usually standardized and less amenable to adaption and tailoring based on the needs, motivation and resources of families in need of treatment. They may therefore be expected to have less strong effects on severe externalizing problems compared to a face-to-face program, which can be individualized to a higher extent by a therapist. In a stepped-care approach^31^, Internet-based interventions could be a first step for families with children with externalizing problems, while group-based or individual face-to-face parent programs are offered as second or third steps, although the latter options may also be directly offered to families with more severe problems and less strengths.

The present study provides an effectiveness trial evaluation, including a 1- and 2-years follow-up, of the Family-Check-Up^32^ compared to the internet-based parent-training program (iComet)^25^, both targeting externalizing behaviors in children. The effects of the more adaptable and tailored intervention FCU compared to a standardized Internet-based parent-training program, such as the iComet, are expected to be significantly larger. It is not meaningful to run another study comparing these treatments to a wait-list control condition. Previous research, as reviewed above, has already shown that they are efficacious in such comparisons. In the face of current needs to provide evidence-based interventions to more families in need of treatment, head-to-head comparison of these interventions would help us know for whom and under what circumstances these interventions may be used in clinical practice. We need to ascertain whether the outcomes for those in various treatment conditions are systematically different in relation to socio-demographics such as child age and parental educational level, or, severity of problems, and motivation for treatment. We also need to understand the processes through which these programs exert their effect, for instance through reduced harsh parenting or improved positive parenting^33^.

Our main research question was to compare the effects of FCU to iComet for children and adolescents (10–13 years old) with conduct problems, on externalizing behaviors, social adaptation, family conflict and warmth, and general psychological health, as reported by themselves, their parents and teachers. We also wanted to evaluate program effects after 1 and 2 years. Clinically reliable changes in externalizing and hyperactivity problems of the FCU and iComet at post-, 1-, and 2-years follow-ups were also examined. To contribute to our understanding of variables systematically affecting the outcome for various subgroups, and potential processes explaining the ways in which the treatments exerted their effects, we also investigated potential moderators (child age, parental education, income level, severity of problems, and parental motivation for treatment) and mediators (parental limit setting, and appropriate or harsh parenting) of outcome. The first hypothesis was that the FCU would more effectively reduce child externalizing problems compared to the iComet, and that the FCU would show large effects on child behavior problems, particularly for those with severe problems, while the iComet would have small to moderate effects. The second hypothesis was that the FCU would show larger effects than iComet on other aspects of family functioning (family conflicts and warmth) and general child psychological health. The third hypothesis was that both programs would have sustained effects at the 1- and 2-years follow-ups, but the maintenance would be greater in the FCU than iComet. The fourth hypothesis was that improvements in parental skills would mediate outcome in both treatments.

## Methods

### Design

The study had an experimental design with randomization to either FCU or iComet and follow-ups after 1 and 2 years. Given the power analysis in the planning phase of the study, a randomization list for 280 participants was prepared. Due to limited resources and time, as well as difficulties recruiting families because of a large number of ongoing parent training studies in Gothenburg, the recruitment (from March 2011 to April 2013) was stopped after including 231 families, which resulted in an uneven number of families in the conditions (FCU: *N* = 122, iComet: *N* = 109). We obtained data from 231 parents, 200 children and 203 teachers at the initial assessment. Despite the uneven numbers of families in the conditions, we found no significant differences or mentionable effect sizes between the conditions on any demographic or baseline measures. The study was approved by the Regional Ethics Review Board in Uppsala (Dnr. 2010/119), and it also was registered at ISRCTN registry, DOI: 10.1186/ISRCTN09352710 (http://www.isrctn.com/ISRCTN09352710. 4/1/ 2013). The research was run in accordance with the ethical guidelines of Swedish Research Council, which mirror those of the American Psychological Association (APA) ethics code. In accordance with criteria and suggestions provided in many reports e.g.^34,35^, the study is characterized as an effectiveness trial. Therapists in regular social services conducted the FCU, there were few exclusion criteria, and the FCU is inherently flexible for adaptations to the needs of the families. It means that some families go on with additional interventions after the check-up, while other families do not receive any further intervention. In addition, one goal was to investigate the subjective outcomes in real-world settings from different perspectives (parents, children and teachers).

### Participants

**Figure 1:**
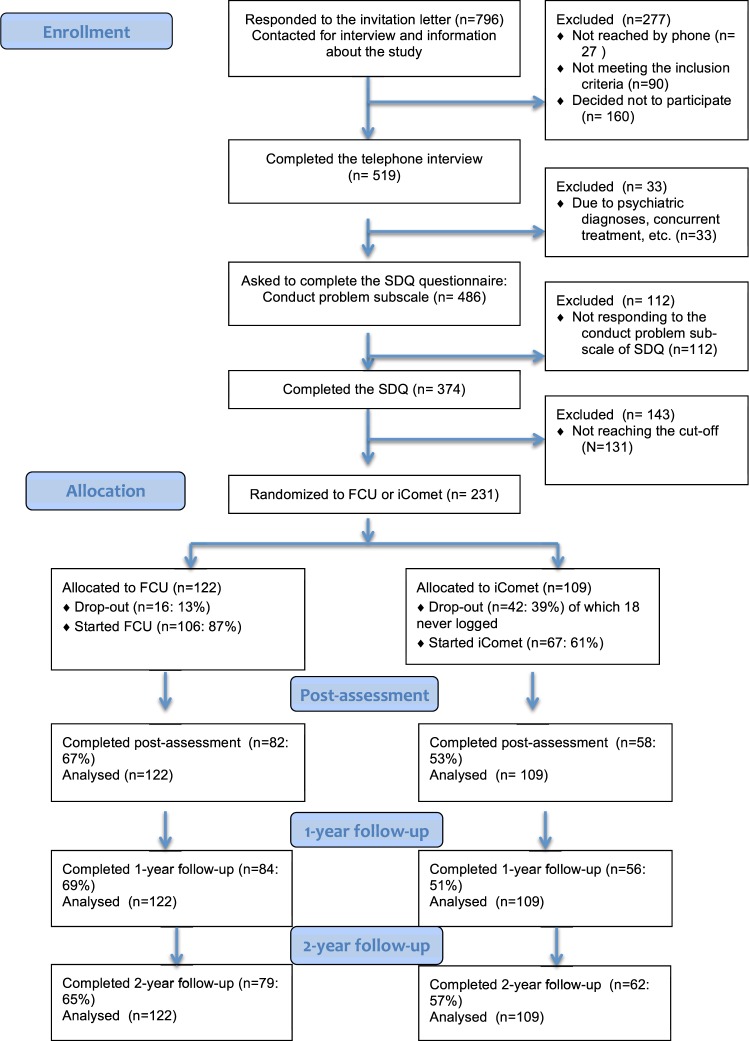
CONSORT flow diagram depicting enrollment, allocation and follow-up.

A total of 231 families with a child aged 10–13 years with conduct problems were enrolled in the study. Figure 1 illustrates the flow of participants into the trial and drop-out until the 2-years follow-up.

The characteristics of the enrolled families, divided into those randomized to FCU versus iComet are shown in Table 1.

**Table 1:** Characteristics of the families in the FCU, iComet, and the total sample.

	FCU *N* = 122	iComet *N* = 109	Total sample *N* = 231
**Parents: Marital status**			
Married	51 (41.8%)	44 (40.4%)	95 (41.1%)
Living together with a partner	35 (28.7%)	32 (29.4%)	67 (29%)
Single parent/divorced	27 (22.1%)	25 (22.9%)	52 (22.5%)
Widowed/other	9 (7.4%)	8 (7.3%)	17 (7.4%)
**Parents: Education**			
Primary school	12 (9.8%)	10 (9.2%)	22 (9.5%)
High school (2 years)	19 (15.6%)	25 (22.9%)	44 (19%)
High school (3–4 years)	26 (21.3%)	27 (24.8%)	53 (22.9%)
College/university	65 (53.3%)	47 (43.1%)	112 (48.5%)
**Family income**			
Insufficient related to expenses	7 (5.7%)	12 (11%)	19 (8.2%)
Almost sufficient	28 (23%)	33 (30.3%)	61 (26.4%)
Sufficient: We don’t worry	71 (58.2%)	57 (52.3%)	128 (55.4%)
Good: Don’t think of expenses	16 (13.1%)	7 (6.4%)	23 (10%)
**Number of children in the family**			
1 child	22 (18.0%)	23 (21.1%)	45 (19.5%)
2 children	59 (48.4%)	47 (43.1%)	106 (45.9%)
3 or more children	41 (33.6%)	39 (35.8%)	80 (34.6%)

A total of 16 participants in the FCU condition (13%) and 24 in the iComet (24%) dropped out after randomization, before the start of intervention. In addition, 18 participants (16.5%) in the iComet did not ever log into the online system. Of those who started the treatment, 22.6% in the FCU and 13.4% in the iComet dropped out before the post-treatment assessment. In total, after randomization, 106 participants (87%) started the FCU intervention, while the corresponding figures for the iComet were 67 (61%). The drop-out was significantly larger in the iComet compared to FCU, and the same drop-out pattern emerged from randomization to post-treatment (*x*^2^ (*1*, *N* = 231) = 4.73, *p* = 0.03). Given the substantial number of drop-outs in both groups, the attrition group (drop-out at post-treatment: *n* = 91) was compared to those completing the trial on the basis of all the demographics and outcome variables at pre-assessment. There were no significant differences between the attrition group and the completers on any of the variables. Because the drop-out rate was significantly different between the conditions, the analyses were redone for each condition separately. No significant differences emerged.

### Procedures

Due to lack of normative data from the general population of parents of children aged 10–13 for the instruments used to measure the outcome of this study and to build a profile as done in the FCU condition, we collected norms from a random sample of the target population in Sweden for details of study to collect norms to be used in the present study see^36^. Next, 13000 invitation letters were sent to parents of children at the ages of 10 to 13, living in the participating districts in the City of Gothenburg. These districts were a mix of those with low, middle and high socioeconomic status. School principals and teachers at grade 4–6 within the participating districts were informed about the study. Information about the study was also presented during school-parent meetings at these schools. Flyers with several different languages other than Swedish were also prepared and distributed at schools and local associations for non-native Swedes. For increased generalizability of the study and inclusion of minorities, we also hired interpreters to provide information at other languages than Swedish. Interpreters also helped during the intervention for families not capable of understanding and reading or writing Swedish. The ads and flyers invited families to participate in the study if they had a child aged 10–13 who showed recurrent signs of disruptive behaviors (oppositional, defiant, or delinquent behaviors such as temper tantrums, generally disobedient, fighting with other children, lying, cheating, or stealing).

Parents interested in participating sent in a letter or e-mail expressing their interest and were then contacted by a research assistant, a graduate, or a doctoral student for more information. A screening interview was then conducted by phone. During this screening, those who were already enrolled in another treatment for their child’s difficulties, those who were planning to move out of the city, or could not accept to be randomized, and those in need of other treatments due to specific conditions such as autism were excluded. After obtaining written informed consent, the parents and the child’s teacher were asked to respond to the Strengths and Difficulties Questionnaire (SDQ)^37^. Children above a cutoff (i.e., three points or more) based on the ratings of parents or their teacher on the SDQ conduct problems subscale^37^ were included in the study. In addition to written information available in several different languages, a professional interpreter explained the content of the consent, when needed. Given the a priori power analysis, a randomization list for 280 participants was prepared. When a family met the criteria for inclusion, and after filling in the pre-assessment battery, the research coordinator contacted the research leader, who informed the coordinator about the condition to which the family should be randomized using the randomization list (generated in Microsoft Word Excel). The research leader (first author) had no information about the family prior to randomization, and the research coordinator was kept blind to the randomization list. A total of 231 parents were finally included and asked to complete the baseline questionnaire on a secure Internet site and randomized into the FCU or iComet. Families were then invited to attend a meeting during which a series of parent-child interactions scenarios were recorded, and then asked to fill in a questionnaire about the characteristics of the neighborhood they live in, and their level of motivation to participate in the trial. Parents who were not fluent in Swedish were invited to respond to the questionnaire in the presence of a research assistant and an interpreter. Children were also asked to respond to a series of questionnaires. To obtain as objective data as possible, the project staff helped children to read through and understand the content of the questionnaires. Thus, data was collected from parents, children and their teachers. The participants had the opportunity to choose between four different gifts after filling in the questionnaires (such as a gift card, two cinema tickets, or donation to charity, all with the same monetary value of approximately 30 USD). The post-intervention assessment (10 weeks after the start) as well as 1- and 2-years follow-ups was completed via the Internet.

### Measures

The internal consistency of all the questionnaires and their subscales (e.g., subscales of the SDQ, etc.) were at least acceptable (>0.70), but in most cases good (>0.80) to excellent.

### Parental ratings

#### Demographics

Parents were asked to provide background information such as educational level, number of children in the household, income level, variables related to their child’s behavior and context (e.g., sleeping habits, smoking, using alcohol or drugs, problems and progress in school) most of which are based on a standardized package of questionnaire specifically adapted for assessment and feedback to parents within FCU.

#### Externalizing behavior problems

This primary outcome variable was measured by Disruptive Behavior Disorders Rating Scale (DBD)^38^ and the SDQ^39^. The DBD covers the DSM-IV-based symptoms of attention deficit/ hyperactivity disorder, oppositional defiant disorder, and conduct disorder. For the present study, we summarized the responses on the DBD using the composite scores, which were calculated by adding the items within each subscale (i.e., oppositional/defiant, inattention and hyperactivity/impulsivity). The DBD possesses good psychometric properties^38^, and its internal consistency (polychoric ordinal alpha) has been shown in our studies of the Swedish version^36^.

#### Conduct, emotional and peer problems

The SDQ^37^ provides the subscales conduct problems, hyperactivity-inattention, peer problems, emotional symptoms and prosocial behavior. The different problem subscales can also be summed into a total difficulties scale. The SDQ has shown good psychometric properties^36,39^.

#### Relationship quality

The short version of the Dyadic Adjustment Scale which possesses good psychometric properties (DAS-4^40^) to assess the quality of relationship between the parents.

#### Family warmth and conflicts

Warmth and conflict in the family consists of five questions on warmth from the Adult-Child Relationship Scale (ACRS)^41^, which is an adaptation of the School-based Student-Teacher Relationship Scale (STRS)^42^ and four questions on conflict adapted from the PAL2 project by the Child and Family Center, University of Oregon, USA.

#### Monitoring

Parental knowledge and monitoring scale (PKMS)^43,44^ assess parenting behaviors where the parent is actively paying attention to and tracking the child’s behaviors and activities. The PKMS provides the subscales parental knowledge, child disclosure, parental solicitation and parental control. Exploratory and confirmatory factor analyses have shown that the child disclosure subscale may best be presented by two constructs, i.e., child disclosure and secrecy^45^. In the present study both these subscales were utilized. The PKMS has good psychometric properties^43,44^.

#### Alcohol and drugs

At baseline and post-assessment, parents were also asked to respond to Alcohol Use Disorders Identification Test (AUDIT)^46^, and to Hospital Anxiety and Depression Scale (HADS)^47^.

#### Moderating variables

The majority of moderators (i.e., child age, parental education, and income level) were obtained via FCU assessment package that was responded to by all the parents). The severity of problems was operationalized by scores on the FCU total difficulties subscale, and the parental motivation to engage in the intervention was measure by the Nijmegen Motivation list-2^48,49^. Finally, we asked parent to rate their perception of crime and disorganization in their neighborhood using the Neighborhood Disorganization^50^.

#### Mediation variables

To measure potential mediation, parents were asked to respond to the following instruments at baseline, five weeks after the start of intervention and at post-treatment: Harsh and inconsistent parenting (15 items) versus Appropriate parenting (12 items), as well as Limit setting (3 items) subscales of the Parenting Practices Interview^51,52^.

#### Child ratings

Children were asked to respond to the SDQ child version^37,39^ (primary outcome measure), child version of the PKMS^43,44^, the Emotion Questionnaire^53^, the Child Depression Inventory-short version^54,55^, child version of the family conflict^41^, and questions about sleeping habits, smoking, alcohol, drugs, prosocial versus deviant peer associations, and problems and progress in school from the FCU child questionnaire. Only the primary outcome reported by children (i.e., child version of the SDQ) will be presented in this study.

#### Teacher-ratings

Teachers were asked to respond to the teacher version of the SDQ^37,39^, teacher version of the DBD^38^, and prosocial versus deviant peer associations as well as problems and progress in school from the FCU-assessment package (teacher check-in). In the present paper, we only present primary outcomes reported by the teacher (the SDQ and DBD).

### Interventions

FCU, described as a second-generation model of parent training, is adapted from the Oregon PMT model combined with techniques from Motivational Interviewing (MI)^56,57^. FCU embrace a first phase with assessment of family strengths and risks summarized in a family profile as a tool for feedback to the family. The next phase is tailored interventions adapted to the family’s needs and motivation. Some families are content with the assessment and feedback phase that encompasses three sessions; brief interview and information about the model, recording of interaction in specific situations between one parent and the child, and one session for feedback of the family profile. Parent training interventions (divided into three areas of skills: supporting positive behavior, setting healthy limits, and building family relationships)^58^ are suggested and chosen depending on needs and motivation emerged during the feedback session. In an extended clinical context other available evidence based interventions can be considered as possible options. There were some adaptations made of the content in the Swedish manual, the most important one being the exclusion of the time-out procedure from the intervention to gain cultural acceptance for the FCU in Sweden, and more heavy focus on antecedents of behavioral problems, clear expectations negotiated between the parent and the child, as well as efficient prompts and when needed both the parent and child taking a break from the acute situation.

The iComet is an adaptation of a Swedish PMT program (Comet)^30^ based on social learning theory/cognitive-behavior therapy. The iComet^25,28^ is provided individually to parents with fewer sessions (7 sessions) compared to Comet (11 sessions) that is implemented in group-format. The major themes covered in iComet are positive parenting, communication, positive reinforcement, while response-cost of problematic child behavior is given less attention. The iComet is a 7-session parent training program, delivered through a secure website under 10 weeks. The program contains text, videos of interactions between a parent and a child, illustrations, and multiple-choice questions about the content of each session. Parents received immediate feedback by the program as to whether the answers they provided were right or wrong, by means of reinforcing statements and explanations. The interaction scenes illustrated both nurturing parent/child interactions with positive reinforcement and positive parenting skills, as well as less optimal interactions. The aim in scenes with less optimal interactions was to stimulate the parents to reflect on what could have been done differently. Each session on the Internet took about 1.5 h to complete for the parent.

### Training

The intervention (FCU) was provided by professionals working within the Swedish Social services, and not by carefully chosen therapist within a university/research setting. Virtually all of those who received training in FCU continued to use it within their usual work context after the end of the trial. Initially both therapists and supervisors had the same introduction in the model by the founders of FCU during four days and then seminars about MI led by an expert in the field during two days. The second author was responsible for training and supervision of both therapists and supervisors. After the initial education, the therapists practiced the use of the model with one or two families each, and received supervision in small groups of therapists with one supervisor after each session. During this period, the therapists also attended regular seminars led by the second author and the supervisors to make sure that all parties are completely clear about all the methods and principles and to share and discuss experiences from meeting the families and supervision. Most therapists had no previous training in parent management models. The supervision was conducted according to the principles discussed with the founders of the FCU, as outlined in the COACH^59^. The supervisors met continuously at least weekly to discuss their supervision of the therapists both in group-settings and individually with the second author. Early in the project period there was a turn-over in the group of supervisors, and some of the therapists were recruited as supervisors. During a shorter period the supervisors received some supervision from the founders of FCU as well. The supervisors also received regular supervision in MI, and they were fully updated about the FCU model by participation in new round of four-day training and seminars, two years after the first occasion. The second author had contact with the founders of the FCU with varied intensity during the project period.

To provide guidance and support for families in the iComet, three Psychology undergraduates (Master of Psychology) were recruited and briefly educated about the basic principles of the reinforcement and how to be supportive for parents to follow the program. A doctoral student within the research project (third author) as well as two research assistants also provided support to the parents. The differences in the level of education and experience were part of the approach to mimic delivery of support in real-world settings. The support that is provided to parents in the iComet condition can be done by non-professionals after receiving basic training on principles of behavior change and general support.

### Fidelity and quality check

All FCU sessions were recorded for later check of fidelity (i.e. adherence and competence). A random sample of 135 sessions (20% of all the sessions) representing all the family therapists and different parts of the intervention were chosen for fidelity ratings using the COACH rating system specifically developed for the FCU^59^. Five categories (conceptual accuracy and adherence; observant and responsive to client needs; actively structures the sessions; careful and appropriate teaching; hope and motivation are generated; family engagement) are rated on a 9-point scale, where 1–3 represents “needs work”, 4–6 “acceptable work”, 7–9 “good work”. A score of 5 shows adequate fidelity to the model. Two independent raters were educated by supervisors within the project and co-rated sessions not included in the fidelity ratings, until they reached high agreement with each other and with supervisor’s ratings of the same sessions. They were then tested by coding 10 other sessions. Their inter-rater agreement on the COACH composite score was 0.87, and the ICC for the rest of the single items of COACH was between 0.35 and 0.91. The main reason for low ICC on some of the items was over-ratings of one of the raters compared to the other rater and the supervisor ratings. These were most obvious on three of the ten sessions. These sessions were thoroughly reviewed and discussed. Then the rater coded five additional sessions on which the ratings were compared to those of the team of the supervisors to achieve good agreement.

To standardize the contact with the iComet families, all the facilitators were instructed to follow a brief manual. All support was delivered through the platform within which all communication with parents was done.

### Statistical analysis

All outcome data were checked for normality using descriptive analyses and Q-Q-plots. As data were collected using an online secure system, item-level missing could not occur, as respondents were asked to provide a rating for each question. The conditions were compared concerning baseline data (demographics and outcome), showing no significant differences. The dropout group was compared to those responding to assessments at different points using analysis of variance, Mann-Whitney U-test, or Chi-2. Descriptive statistics such as mean, standard deviation, or median were used when needed to illustrate different parameters. To assess inter-rater reliability, Intraclass correlations (ICC) based on a two-way mixed effect model were calculated.

To study the outcome, we used generalized linear mixed effects regression modeling (GLMM) in IBM SPSS statistics 23. As the GLMM uses maximum likelihood estimation, all the participants were included in the analyses, regardless of having full or partial data (due to drop-out). The advantages of GLMM over more traditional approaches are many, but the most important ones are the ability to include all the participants, and customizing the covariance structure, and other distributions than normal to reflect the nature of data. To examine specific groupXtime effects for the intervention, and the follow-up periods respectively, we performed piecewise regression models^60^. Piecewise regression separating the intervention period from the follow-up period provides a more logical basis for investigating the outcome than considering all the time points as following the same growth rate. A random intercept for individuals and normal or poisson covariance structure with identity or log links dependent on the distribution of data was specified in the analyses. Specification of additional elements such as random slope did not lead to significantly better fit. For multiple comparisons, we applied Sidak corrections (presenting only the significant ones in the Results), and we used Cohen’s *d* to determine the magnitude of the effects.

Potential moderation was also investigated using GLMM (piece-wise regression models) by adding the moderator and its interaction with the independent variable to the model.

Mediators were measured halfway through the intervention. To assess mediation, we used the Process (version 2.16) for SPSS^61^. Process uses ordinary least squares or when required a logistic regression-based path analytic framework to estimate the indirect and direct effect in mediation models. To make inferences about the indirect effects and the effect sizes, the Process also implement bootstrapping and Monte Carlo confidence intervals.

## Results

### Equality of conditions at baseline and engagement

We obtained data from 231 parents, 200 children and 203 teachers at the initial assessment. We found no significant differences between the conditions on any demographic or baseline measures. Of the 106 families who engaged in the FCU intervention (out of 122 who were randomized into the FCU), twenty-two families (20.8%) received only the assessment part of the FCU given the outcome of the assessment and the needs and motivation of the family, while the rest (*n* = 84, 79.2%) also received the parent training intervention part of the FCU (with a mean number of 5.45 (*SD* = 3.1) attended sessions, ranging from two to 22 sessions) in addition to the three initial assessment sessions. Of the 109 families randomized to the iComet, 67 families (61%) engaged in the intervention. The mean number of completed sessions and tasks (out of 15) was 7.7 (*SD* = 5.0), and the median as well as mode was 7 and 15 respectively.

### Fidelity ratings

The COACH rating system yields mean values between 1 and 9 for each of the subscales, with 1–3 indicating need of further work, 4–6 as competent work, and 7–9 as excellent work. The COACH ratings of the selected session resulted in the following mean values and standard deviations: Conceptual accuracy and adherence (*M* = 5.4, *SD* = 0.88), Observant and responsive to client needs (*M* = 5.3, *SD* = 0.99), Actively structures the sessions (*M* = 5.0, *SD* = 1.1), Careful and appropriate teaching (*M* = 5.0, *SD* = 0.87), Hope and motivation are generated (*M* = 4.9, *SD* = 0.88), Family engagement (*M* = 5.8, *SD* = 1.2), and COACH composite score (*M* = 5.2, *SD* = 0.79). These all represent adequate fidelity ratings^59^.

### Short-term (pre- to post-assessment) effects of primary outcome variables

After exploring data using descriptive analyses and Q-Q plots, we specified a normal distribution and an identity link for variables showing fairly normal distribution. For highly positively skewed data, such as the DBD, we specified a gamma distribution and a log link in the GLMM analyses to get the best fit with least complexity. Means and standard errors of all the primary outcome variables for each condition and time points are presented in Table 2.

**Table 2:** Mean and standard error of the parent- and child-reported main outcome variables for the FCU (*N* = 122) and iComet (*N* = 109) at each time point in a GLMM model with all the time points in the same GLMM model.

	Family check-up				iComet			
	Pre-treatment	Post-treatment	1-year FU	2-year FU	Pre-treatment	Post-treatment	1-year FU	2-year FU
**Parent ratings**								
DBD subscales:								
Oppositional/Defiant	1.50 (0.062)	1.07 (0.065)	1.07 (0.064)	1.01 (0.066)	1.50 (0.069)	1.23 (0.076)	1.19 (0.076)	1.12 (0.076)
Inattention	1.29 (0.073)	1.15 (0.076)	1.04 (0.075)	1.04 (0.076)	1.51 (0.081)	1.28 (0.087)	1.28 (0.087)	1.14 (0.086)
Impulsivity/Overactivity	0.96 (0.075)	0.73 (0.059)	0.63 (0.051)	0.60 (0.049)	0.98 (0.084)	0.80 (0.074)	0.76 (0.070)	0.73 (0.068)
SDQ-total difficulties	16.25 (0.603)	12.39 (0.625)	11.77 (0.617)	11.05 (0.630)	16.80 (0.666)	13.45 (0.722)	12.26 (0.726)	13.18 (0.724)
Conduct problems	4.30 (0.215)	2.72 (0.150)	2.55 (0.136)	2.38 (0.133)	3.99 (0.220)	3.07 (0.198)	2.72 (0.184)	2.61 (0.172)
Hyperactivity/Inattention	5.58 (0.262)	4.86 (0.271)	4.40 (0.268)	4.33 (0.272)	5.77 (0.289)	5.07 (0.312)	4.71 (0.314)	4.83 (0.312)
Peer problems	2.57 (0.203)	2.19 (0.214)	2.27 (0.210)	1.85 (0.215)	2.81 (0.224)	2.57 (0.252)	2.40 (0.252)	2.61 (0.249)
Emotional symptoms	3.58 (0.247)	2.59 (0.194)	2.73 (0.208)	2.57 (0.195)	3.87 (0.295)	2.51 (0.217)	2.45 (0.218)	3.04 (0.264)
Prosocial behavior	6.57 (0.216)	7.16 (0.226)	7.26 (0.223)	7.4 (0.228)	6.61 (0.239)	7.45 (0.263)	7.09 (0.265)	7.06 (0.265)
**Child ratings**								
SDQ-total difficulties	11.97 (0.566)	12.48 (0.634)	11.67 (0.638)	12.93 (1.425)	11.89 (0.620)	11.94 (0.685)	12.69 (0.666)	12.07 (2.022)
Conduct problems	2.41 (0.165)	2.55 (0.189)	2.23 (0.189)	2.48 (0.447)	2.56 (0.180)	2.49 (0.204)	2.49 (0.196)	2.46 (0.634)
Hyperactivity/Inattention	4.78 (0.246)	4.83 (0.277)	4.88 (0.277)	4.74 (0.616)	4.52 (0.270)	4.56 (0.299)	5.08 (0.289)	5.3 (0.873)
Peer problems	2.08 (0.168)	2.20 (0.191)	1.99 (0.193)	2.20 (0.458)	1.94 (0.184)	1.94 (0.206)	2.08 (0.200)	1.99 (0.653)
Emotional symptoms	2.72 (0.184)	2.97 (0.230)	2.54 (0.200)	3.21 (0.531)	2.89 (0.215)	2.84 (0.237)	2.95 (0.237)	2.49 (0.577)
Prosocial behavior	7.84 (0.189)	7.90 (0.213)	7.70 (0.217)	6.83 (0.513)	8.43 (0.207)	7.71 (0.230)	7.97 (0.224)	7.92 (0.731)

DBD: Disruptive Behavior Disorders Rating Scale, SDQ: Strengths and Difficulties Questionnaire.

The first main outcome variable in the study was the DBD. Repeated measure GLMM for parent reported oppositional/defiant subscale of the DBD showed a significant time effect (*F*(1, 588) = 69.70, *p* = 0.001, *d* = 1.10), and tendency toward a significant interaction (Table 3). For the inattention subscale of the DBD, we found a significant time effect (*F*(1, 588) = 19.26, *p* = 0.001, *d* = 0.58), but no interaction. For the hyperactivity/impulsivity subscale, we found a significant time effect (*F*(1, 554) = 27.73, *p* = 0.001, *d* = 0.69), but no interaction.

**Table 3:** GLMM-based Interaction effect (groupXtime) from pre-to post assessment (*N* = 231), and during the follow-up period, respectively for the primary outcome variables reported by parents.

	*Pre- to post-assessment*	*Post-assessment to 1- and 2-years follow-up*
**DBD subscales:**		
Oppositional Defiant	*F*(1, 588) = 3.45, *p* = 0.06, *d* = 0.24	*F*(2, 588) = 0.14, *p* = 0.87, *d* = 0.05
Inattention	*F*(1, 588) = 1.31, *p* = 0.25, *d* = 0.15	*F*(2, 588) = 1.45, *p* = 0.24, *d* = 0.16
Impulsivity/Overactivity	*F*(1, 554) = 0.52, *p* = 0.47, *d* = 0.09	*F*(2, 554) = 0.71, *p* = 0.50, *d* = 0.11
**SDQ subscales:**		
SDQ-total difficulties	*F*(1, 583) = 0.47, *p* = 0.49, *d* = 0.09	*F*(2, 583) = 2.35, *p* = 0.09, *d* = 0.20
Conduct problems	*F*(1, 540) = 5.22, *p* = 0.02, *d* = 0.30	*F*(2, 540) = 0.18, *p* = 0.84, *d* = 0.06
Hyperactivity/Inattention	*F*(1, 583) = 0.02, *p* = 0.92, *d* = 0.02	*F*(2, 583) = 0.31, *p* = 0.73, *d* = 0.07
Peer problems	*F*(1, 583) = 0.23, *p* = 0.63, *d* = 0.06	*F*(2, 583) = 1.98, *p* = 0.14, *d* = 0.19
Emotional symptoms	*F*(1, 483) = 1.10, *p* = 0.30, *d* = 0.14	*F*(2, 483) = 3.07, *p* = 0.04, *d* = 0.23
Prosocial behavior	*F*(1, 583) = 0.74, *p* = 0.39, *d* = 0.11	*F*(2, 583) = 1.66, *p* = 0.19, *d* = 0.17

DBD: Disruptive Behavior Disorders Rating Scale, SDQ: Strengths and Difficulties Questionnaire.

**Figure 2:**
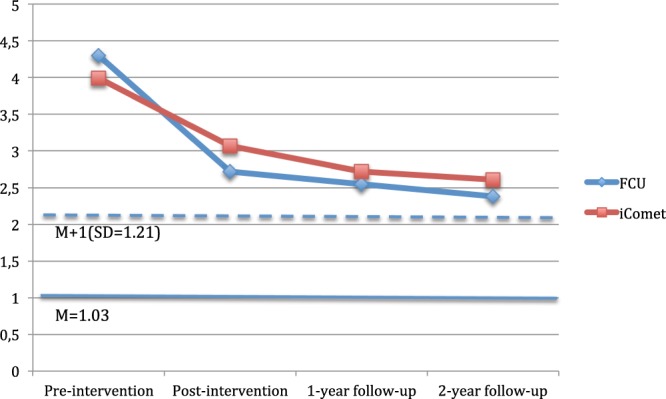
Changes in the conduct problem subscale of the Strengths and Difficulties Questionnaire across time. The mean and +1 SD of the general population is shown by horizontal solid and dashed line to provide a context.

The second main outcome variable was the SDQ. Parent-reported SDQ total difficulties showed a significant time effect (*F*(1, 583) = 96.17, *p* = 0.001, *d* = 1.29), but no significant interactions between the FCU and the iComet across time (Table 3). For the SDQ conduct problems, we found a significant time effect (*F*(1, 540) = 70.11, *p* = 0.001, *d* = 1.10), and a significant interaction (*F*(1, 540) = 5.22, *p* = 0.02, *d* = 0.30) as seen in Table 3, and Fig. 2. The mean reduction of the conduct problems reported by parents from pre- to post-assessment was significantly larger in the FCU compared to the iComet condition. For hyperactivity and inattention, we found a significant time effect (*F*(1, 583) = 21.49, *p* = 0.001, *d* = 0.61), but no significant interaction. There was also a time effect for peer problems (*F*(1, 583) = 4.11, *p* = 0.04, *d* = 0.27), but no significant interaction. Further, parent-rated emotional symptoms significantly decreased from pre-to post-treatment (*F*(1, 483) = 53.44, *p* = 0.001, *d* = 0.96), but no significant interaction emerged. In terms of parent-reported child strengths, the prosocial behaviors significantly increased from pre- to post-intervention (*F*(1, 583) = 24.44, *p* = 0.001, *d* = 0.65), but we found no significant interaction.

With the exception of the prosocial subscale, none of the subscales of the child-reported SDQ showed a significant time, group or interaction effects. The estimated means and standard errors are presented in Table 2. We found a significant time effect (*F*(1, 439) = 4.43, *p* = 0.04, *d* = 0.32) on the prosocial subscale, and a significant interaction (*F*(1, 439) = 6.17, *p* = 0.01, *d* = 0.37) due to a certain decrease in the iComet condition from pre- to post-treatment compared to the FCU.

For the teacher-reported DBD and SDQ, we found no significant effects at all from pre-to post-treatment. The corresponding effect sizes were virtually non-existent or very small to justify detailed report of the analyses.

### Long-term outcome of primary outcome variables: from post- to 1- and 2-years follow-up

During the follow-up period, the oppositional/defiant subscale of the DBD remained unchanged, reflected in a non-significant time effect (*F*(2, 588) = 1.58, *p* = 0.21, *d* = 0.17), and a non-significant interaction (Table 3). For the inattention subscale of the DBD, we found a significant time effect during the follow-up (*F*(2, 583) = 3.62, *p* = 0.04, *d* = 0.25) reflecting a weak linear decrease across the time points, but no interaction. The same pattern emerged for the impulsivity/overactivity subscale, with a significant time effect (*F*(2, 554) = 3.72, *p* = 0.03, *d* = 0.25), but no significant interaction. Means and standard errors are shown in Table 2.

For the parent reported SDQ total difficulties during the follow-up period, we found a tendency toward a significant time effect (*F*(2, 583) = 2.82, *p* = 0.06, *d* = 0.22), indicating a small decrease from post-assessment to 2-years follow-up for both programs, but no significant interaction (Table 3). For the conduct problems subscale of the SDQ, we found a significant time effect (*F*(2, 540) = 4.42, *p* = 0.01, *d* = 0.28) with some further decrease in conduct problems, but no significant interaction, and the same patterns emerged for the hyperactivity/inattention (i.e., a significant time effect (*F*(2, 583) = 3.49, *p* = 0.03, *d* = 0.25), and not interaction). For peer problems, we found neither a time (*F*(2, 583) = 0.37, *p* = 0.69, *d* = 0.08), nor an interaction effect (Table 3). Although emotional symptoms did not show a significant time effect during the follow-up period (*F*(2, 483) = 1.42, *p* = 0.24, *d* = 0.16), we found a significant interaction (Table 3), which was due to stable outcome in the FCU from 1- to 2-years follow-up in contrast to a slight deterioration in iComet during the same period. However, the differences between the conditions at the last follow-up time point was only marginally significant in Sidak corrected pairwise comparisons (*t*(483) = 1.73, *p* = 0.08). For parent-reported prosocial behavior during the follow-up period, we found neither a time effect (*F*(2, 583) = 0.40, *p* = 0.67, *d* = 0.08), nor any interactions.

For the child reported SDQ total difficulties scores during the follow-up period, we did not find any effects at all. Lack for significant effects was also evident in teacher-reported main outcome during the follow-up period.

### Short-term effects of secondary outcome variables

We found a significant time effect for family warmth (*F*(1, 647) = 34.95, *p* = 0.001, *d* = 0.78), reflecting an increase from pre- to post-treatment, but no interaction (*F*(1, 647) = 0.02, *p* = 0.88, *d* = 0.02). Mean and standard errors for all the secondary outcomes are presented in Table 4. The same pattern emerged for family conflict: a significant time effect (*F*(1, 647) = 12.00, *p* = 0.001, *d* = 0.46), but no interaction (*F*(1, 647) = 0.18, *p* = 0.84, *d* = 0.06). With regard to the quality of the relationship between the parents and their partners, measured by the DAS-4, we collected data up to the 1-year follow-up, and dropped the measurement from the 2-years follow-up to reduce response burden. We found no significant time or interaction effect from pre- to post-treatment (*F*(1, 508) = 1.09, *p* = 0.30, *d* = 0.14, and *F*(1, 508) = 0.33, *p* = 0.57, *d* = 0.08).

**Table 4:** Mean and standard error of the parent- reported secondary outcome variables for the FCU and iComet at each time point in a GLMM model with all the time points in the same GLMM model.

	*FCU* (N = 122)				*iComet* (N = 109)			
	*Pre-treatment*	*Post-treatment*	*1-year FU*	*2-year FU*	*Pre-treatment*	*Post-treatment*	*1-year FU*	*2-year FU*
Family warmth	19.04 (0.326)	20.82 (0.362)	20.23 (0.364)	19.58 (0.373)	18.92 (0.345)	20.27 (0.412)	19.76 (0.427)	19.21 (0.424)
Family conflict	8.44 (0.446)	6.90 (0.506)	6.61 (0.504)	6.53 (0.516)	8.13 (0.427)	7.20 (0.582)	6.85 (0.599)	6.38 (0.587)
Dyadic Adjustment Scale	10.61 (0.701)	10.02 (0.744)	11.35 (0.757)	No data*	11.05 (0.741)	10.87 (0.824)	10.55 (0.864)	No data*
**PKMS subscales:**								
Parental Knowledge	1.78 (0.048)	1.72 (0.051)	1.75 (0.053)	1.83 (0.057)	1.72 (0.050)	1.67 (0.056)	1.67 (0.059)	1.82 (0.065)
Parental Solicitation	2.69 (0.067)	2.69 (0.075)	2.27 (0.064)	2.25 (0.065)	2.62 (0.069)	2.57 (0.082)	2.21 (0.74)	2.3 (0.76)
Secrecy	3.88 (0.081)	4.06 (0.095)	3.99 (0.095)	3.97 (0.098)	3.87 (0.086)	4.04 (0.108)	3.93 (0.111)	4.00 (0.113)
Child Disclosure	2.55 (0.087)	2.31 (0.097)	2.23 (0.098)	2.32 (0.101)	2.65 (0.092)	2.47 (0.111)	2.37 (0.116)	2.46 (0.115)
Parental Control	1.32 (0.044)	1.27 (0.048)	1.37 (0.052)	1.58 (0.062)	1.28 (0.048)	1.29 (0.056)	1.36 (0.062)	1.49 (0.066)

PKMS: Parental Knowledge and Monitoring Scale.

*No data: We did not include Dyadic Adjustment Scale in the 2-years follow-up to decrease response burden.

The parental knowledge subscale of the PKMS showed neither a time (*F*(1, 651) = 2.62, *p* = 0.11, *d* = 0.21) nor an interaction effect (*F*(1, 651) = 0.06, *p* = 0.81, *d* = 0.03). The pattern was similar for parental solicitation, with neither a time effect (*F*(1, 651) = 0.32, *p* = 0.57, *d* = 0.07), nor an interaction (*F*(1, 651) = 0.20, *p* = 0.66, *d* = 0.06). For the secrecy subscale, we found a significant time effect (*F*(1, 651) = 7.78, *p* = 0.005, *d* = 0.37), but no significant interaction (*F*(1, 651) = 0.90, *p* = 0.77, *d* = 0.12). The outcome was similar for disclosure, showing a time effect (*F*(1, 651) = 9.65, *p* = 0.002, *d* = 0.41), but no interaction (*F*(1, 651) = 0.22, *p* = 0.64, *d* = 0.06). Finally, for parental control, we found neither a time (*F*(1, 651) = 3.69, *p* = 0.06, *d* = 0.25), nor an interaction effect (*F*(1, 651) = 0.26, *p* = 0.61, *d* = 0.07).

### Long-term effects of secondary outcome variables

Across the follow-up period, there was significant time effect for family warmth (*F*(2, 647) = 8.28, *p* = 0.88, *d* = 0.38), but no significant interaction (*F*(2, 647) = 0.05, *p* = 0.95, *d* = 0.03). Neither a time, nor an interaction effect was found for family conflict during the follow-up period, and a similar pattern emerged for DAS-4, with no change from post-treatment to 1-year follow-up (*F*(1, 508) = 2.44, *p* = 0.12, *d* = 0.21), and no significant interaction (*F*(1, 508) = 2.35, *p* = 0.13 *d* = 0.20). Parental knowledge subscale of the PKMS showed a significant time effect during the follow-up period (*F*(2, 651) = 6.55, *p* = 0.002, *d* = 0.34), which was due to a general increase from 1- to 2-years follow-up period, but no significant interaction (*F*(2, 651) = 0.39, *p* = 0.68, *d* = 0.08). Similarly, we found a significant time effect for parental solicitation during the follow-up (*F*(2^1^, 651) = 30.68, *p* = 0.001, *d* = 0.73) due to a large decrease in parental solicitation from post-treatment to 1-year follow-up. However, there was no interaction for parental solicitation (*F*(2, 651) = 1.14, *p* = 0.32, *d* = 0.14). For secrecy, there was no significant time (F(2^1^, 651) = 1.65, *p* = 0.19, *d* = 0.17) or interaction effect (*F*(2, 651) = 0.16, *p* = 0.86, *d* = 0.05) during the follow-up. The same pattern emerged for disclosure with no time (F(2, 651) = 0.22, *p* = 0.64, *d* = 0.06) or interaction effect (*F*(2, 651) = 0.01, *p* = 0.99, *d* = 0.04). For parental control, however, we found a significant time effect (*F*(2, 651) = 0.42, *p* = 0.66, *d* = 0.52), but no significant interaction (*F*(2^1^, 651) = 9.65, *p* = 0.002, *d* = 0.09).

### Clinically significant change

The proportion of participants in each condition who made a reliable change on the main outcome variables (“improved”), as well as a conservatively defined clinically significant change on the main outcome variables (i.e., those who both made a reliable change and ended up with a value within one standard deviation of mean of the general population, called “recovered”) is presented in Table 5.

**Table 5:** The percentage of participants in each condition (FCU = 122, iComet = 109) who recovered (i.e., both made a reliable change and a clinically significant change in terms of transferring into values within one standard deviation of the mean of the general population), improved (i.e., made a reliable change), unchanged, or deteriorated (i.e., made a reliable change in the undesired direction) based on the main outcomes at different assessment points for each condition.

	Pre- to post-assessment		Pre- treatment to 1-year FU		Pre- treatment to 2-year FU	
	FCU	iComet	FCU	iComet	FCU	iComet
**DBD: Oppositional Defiant**						
Recovered	**18**.**0**^**a**^	**6**.**4**^**a**^	**26**.**2**^**b**^	**11**.**0**^**b**^	36.1	25.7
Improved	53.3	60.6	48.4	60.6	40.9	46.8
Unchanged	20.5	17.4	10.6	17.4	8.2	17.4
Deteriorated	8.2	15.6	14.8	11.0	14.8	10.1
**DBD: Inattention**						
Recovered	**18**.**9**^**c**^	**9**.**2**^**c**^	32.8	30.3	27.0	27.5
Improved	19.6	30.2	28.7	38.5	**32**.**8**^**d**^	**48**.**6**^**d**^
Unchanged	42.6	46.8	18.8	11.9	23.8	8.3
Deteriorated	18.9	13.8	19.7	19.3	16.4	15.6
**DBD: Impulsivity/hyperactivity**						
Recovered	13.1	11.0	34.4	32.1	**36**.**1**^**e**^	**21**.**1**^**e**^
Improved	47.6	55.1	35.3	36.0	38.5	45.0
Unchanged	22.9	18.3	18.0	13.6	12.3	14.6
Deteriorated	16.4	15.6	12.3	18.3	13.1	19.3
**SDQ: Total difficulties**						
Recovered	16.4	13.8	53.3	60.6	41.0	36.7
Improved	17.2	13.7	7.4	5.5	24.6	23.9
Unchanged	65.6	69.7	38.5	30.2	31.9	33.9
Deteriorated	0.8	2.8	0.8	3.7	2.5	5.5
**SDQ: Conduct problems**						
Recovered	**27**.**9**^**f**^	**11**.**0**^**f**^	33.6	29.4	43.4	34.9
Improved	33.6	35.8	23.0	33.9	29.6	30.2
Unchanged	36.9	51.4	42.6	33.0	26.2	34.0
Deteriorated	1.6	1.8	0.8	3.7	0.8	0.9

DBD: Disruptive Behavior Disorders Rating Scale, SDQ: Strengths and Difficulties Questionnaire.

Comparisons with the same superscripts within each row are significantly different (χ^2^(1, N = 231) = 3.99 to 10.26, *p* = 0.036 to 0.001. If family-wise correction is applied, only comparisons marked with “b” and “f” remain statistically significant.

As shown in Table 5, which is based on parent reports, a significantly larger proportion of children in the FCU recovered compared to the iComet both from pre- to post-treatment and at 1-year follow-up with regard to oppositional defiant behaviors as defined by the DBD. Although this pattern was seen at 2-years follow-up as well, the difference was not statistically significant.

At post-assessment a larger proportion of children in the FCU was classified as recovered in terms of inattention compared to iComet, but this difference was not maintained at 1-, and 2-years follow-up. On the other hand, at the 2-year follow-up a significantly larger proportion of families in the iComet were categorized as improved. In a similar way, a non-significantly larger portion of children in the FCU were classified as both improved and recovered in terms of conduct problems (conduct problem subscale of the SDQ) at post-intervention, compared to the iComet. The proportion of children classified as recovered from hyperactivity and impulsivity and (DBD) was also higher in the FCU compared to the iComet at 2-years follow-up. A consistent finding was a larger proportion of children classified as deteriorated (i.e., they did a reliable change, but in the wrong direction) in the iComet compared to the FCU. However, in none of these instance the difference was statistically significant.

### Moderation and mediation analysis

Neither children’s age, nor parents’ education (dichotomized into high or low), or income significantly moderated the effect of FCU versus iComet. Before starting the intervention, parents were also asked to respond to Nijmegen Motivation List-2^48^ and characteristics of the neighborhood they lived in. Their motivation to participate and engage in the intervention, and neighborhood disorganization was separately entered into the moderation analysis, but they were neither a significant predictor, nor a significant moderator of the main outcome effects. The parent-rated initial severity of the externalizing problems we not a significant moderator either. None of the hypothesized mediator turned out to be significant, and the change in mediators from pre- to mid-treatment was small and insignificant.

## Discussion

The present study examined the relative impact of the FCU and iComet in an effectiveness randomized controlled trial (RCT) with a multi-assessment strategy including parents, children and teachers, evaluating short- and long-term effects on child well-being, in particular conduct problems, and family functioning. The results indicated that both programs had positive effects directly after treatment and after 1 and 2 years according to parent-ratings, but not child- or teacher-ratings. Interestingly, even though the FCU include face-to-face family-meetings with a therapist and the iComet is built upon self-help where parents mainly work independently with therapeutic principles, parents generally reported both programs to be effective in improving child and adolescent psychological health. We also found some essential differences between the programs that might have importance for implementation and clinical decision-making. These are discussed in some more detail below.

Both programs showed short-, and long-term effects on the main outcome variables: conduct problems (large effect sizes), inattention (moderate effect sizes) and impulsivity/hyperactivity problems (moderate effect sizes). These results are similar to those in earlier studies of the programs with parent-reported effects on child conduct problems. As shown in Fig. 2, both intervention groups approached a conservative criterion of falling within one standard deviation of the mean of the normal population, with those in the FCU making a significantly larger change from pre- to post-intervention compared to those randomized to the iComet. Interestingly, both conditions worked out equally well during the follow-up period, as indicated by the virtually parallel lines in Fig. 2. The FCU has been evaluated in several RCT-studies targeting various populations with very encouraging results^7,20,32^, whereas the iComet has been evaluated in one RCT only^25^. Parents allocated to the iComet in the Enebrink *et al*. (2012) study were compared with a waitlist control group and followed-up after 18-months, showing similar effects as face-to-face PMT-treatments^25,28^. Importantly, even though both programs showed reductions in conduct problems in the present trial, there were some additional improvements for parents participating in the FCU. Parents allocated to the FCU reported larger decreases in mean on conduct problems (SDQ subscale) than those allocated to the iComet (but not when evaluating the oppositional/defiant subscale of DBD). This finding shows a small tendency towards the FCU improving conduct problems more than the iComet between the pre- and post-measurement. At the same time, there were continued reports of reductions in conduct problems (but not inattention or impulsivity/hyperactivity) for parents in both programs during the 1- and 2-years follow-ups, and we found no significant mean differences between the programs at the 2-years follow-up. Further, according to the clinical reliable change index, the FCU encompassed a larger proportion of children that were recovered in oppositional defiant behavior, conduct problems, and inattention at the post measurement. These differences were not maintained at the 2-years follow-up, but parents allocated to the FCU reported a larger proportion of recovered children from hyperactivity/impulsivity. Thus, there are tendencies for the FCU to improve conduct problems more than the iComet, particularly directly after treatment.

Parents (but not children or teachers) also reported enhancements on several of the secondary outcome variables in the expected directions both for the FCU and the iComet (emotional symptoms, peer problems, prosocial behaviors, child secrecy and disclosure, family warmth and family conflict). Neither the FCU nor the iComet significantly improved parental knowledge, parental solicitation, parental control, or the quality of the relationship between the parents. The reported secondary variables are important in the context of conduct problems and child well-being for continued risk reduction. For instance, earlier research has suggested that it is when the adolescent him- or herself wants to tell the parents about his or her whereabouts (e.g., child disclosure) that parental knowledge is improved and risk for future behavior problems may be further reduced^44^. Further, in a longitudinal study, Dishion and colleagues (2010)^62^ showed that cascading peer dynamics might contribute to the progression of problem behavior to violence, thus indicating the importance of improving the peer context during early adolescence. A final example includes a trial with the FCU evaluating family conflict in the prevention of adolescent depression^21^. Families with children in middle school, randomized to the FCU reported less increases in child depressive symptoms and family conflicts from 6^th^ to 9^th^ grade. In families with increased family conflicts, an enhanced risk for later depressive symptoms was evident. In terms of generalizability of the findings, it should be kept in mind that the participating parents had a higher level of education than the general population, although families were recruited from several parts of the Gothenburg, some with middle/high, and some with low/very low socioeconomic status.

The development of an internet-based PMT-program was motivated by the needs to reach more families with evidence-based therapies with novel and multiple models of delivery^63^ and to reduce barriers for participation through improved accessibility. Psychological treatments translated into web-based programs have increasingly been shown to have similar effects as face-to-face treatments^64^, and are suggested a possible way forward for transporting evidence-based therapies to rural areas. When translating psychological programs into a more self-administered format it is imperative to understand for whom and when it works, and for whom a face-to-face program should be recommended instead. We did not find any moderators that could guide decision-making, probably due to the similar outcomes and parallel processes. It should be noted that both interventions are based on the Oregon model of parent training, and have a lot in common, although the FCU is a more advanced intervention. In terms of the processes through which the interventions exert their effect, none of the hypothesized mediators turned out to be significant. Increase in appropriate parenting as a consequence of more time devoted to interaction between child and parent, and decrease in harsh parenting were two of several mediators that were investigated mid-way through the treatment. Not only they were not a mediator of outcome, but also the magnitude of change in these mediators from pre to mid-treatment was also low. This was true for all the mediators, which may be due to low sensitivity of the chosen measures, the time frame, or genuine lack of significant and meaningful changes.

Notably, a larger number of parents dropped out from the iComet after being randomly allocated to this program and not to the FCU. Since 16.5% in the iComet condition never logged in to the program, this finding might have to do with treatment expectations and an appeal among the families to meet with a therapist and not completing the treatment online, even though the motivation for obtaining treatment in general before the randomization was similar. When employing an internet-based program in effectiveness trials, it could be important to investigate participant expectations^65^ and how various requests could be met, so that family incentives for following through with the treatment are not reduced.

The FCU has earlier been evaluated in the USA. In the current study, the implementation process for the FCU worked well with some adaptations of the program content. The present study adds to the growing literature about the transportability and implementation of PMT programs. Gardner and colleagues (2016)^66^ conducted a meta-analysis of established parenting programs for parents of children aged 3–10 that had been examined in another country from where it was developed (i.e., the Incredible Years, PMTO, Triple P and the Parent Child Interaction Therapy). Their results indicated that these parenting programs worked equally well when implemented into a new country as in the original setting. Extensive adaptation did not seem necessary. The PMT programs seemed to work even better in foreign countries and cultures. This finding is however not axiomatic for psychological treatments. Sundell and colleagues (2015)^67^ compared general psychological and social interventions in Sweden and preventative interventions in Germany, and found that adopted programs without any adaptation seemed less effective than novel or adapted programs. These systematic reviews differ in targeted age groups, type of programs that were included and outcomes evaluated. Together the reviews demonstrate the importance in evaluating programs when transferring these into a new context and culture. Understanding whether an adopted program needs to be adapted or not to be most effective will improve decision-making for families, therapists, and policy-makers.

### Limitations

The results should be interpreted in context of its limitations. In the present study we aimed for a multi-assessment strategy, including children, parents, and teachers. However, due to the large number of new teachers across the time and their reported lack of knowledge about the children to rate according to DBD and the SDQ, the reliability and validity of long-term results based on teachers’ report are highly uncertain. Inherent difficulties in adequate statistical modeling of different teachers reporting the outcome at different time points during the follow-up is another factor that calls for cautious interpretation of non-significant teacher-reported outcomes. The iComet had a larger drop-out among the families than the FCU. This may to some extent be attributable to the overall study-information that parents would be randomized either to therapist or internet-based support, and some parents may have built a preference for any of the programs. At the time of the study, internet-based parenting support was not widely spread, which might have contributed to a possible lack of parental interest. It would have been easier to understand the drop-out if a measure of credibility and expectancy had been delivered at an early stage. However, due to high response burden, such a measure was not included. The early drop-out in the FCU was fairly low (13%). Non-participation in the post-intervention assessment was most probably a consequence of an extensive and demanding assessment procedure, requiring the parents and their children to respond to a large number of questionnaires. Decreasing the response burden should be a priority in future effectiveness studies. Another limitation of the study is its reliance on self-report measures. The study could have included a waitlist as a third condition, to draw firmer conclusions about the effectiveness of the interventions. However, when the efficacy of interventions compared to a wait-list control or placebo condition has been established, head-to-head comparison and factorial designs provide more useful information, especially when the comparisons are within the frame of effectiveness. Finally, the originally intended total sample size, based on power analysis was 280, while we only managed to enroll 231 families. Lack of significant effects might be a consequence of smaller than intended sample size. The reported effect sizes, regardless of *p*-values, provide a valuable source to make more informative calculation of power for future head-to-head comparisons.

## Conclusion

To conclude, this effectiveness trial of FCU and iComet showed that both parenting programs reduced child conduct problem behaviors, as well as inattention, impulsivity and hyperactivity problems. The parent ratings also showed that peer problems and emotional symptoms decreased and prosocial behaviors increased from pre- to post-treatment assessment. These improvements were generally either retained or continued to improve to the 2-years follow-up. At the 2-years follow-up, the majority of the children had further decreased their conduct problem behaviors, and many of them had made a clinically significant change, particularly those in the FCU. The FCU and iComet might be employed in a stepped-care context, or depending on family interests and possibility for participating in face-to-face or internet-based treatments. The results in the context of previous findings suggest further evaluation and cautious implementations of this extended family of PMT-programs, developed for encompassing the whole family (FCU) or for being administered and available through the Internet (iComet).
